# Delayed presentation of a non-resorbing postpartum vulvar hematoma: A case report

**DOI:** 10.1016/j.crwh.2023.e00518

**Published:** 2023-06-07

**Authors:** Jeremy Winkelman, Kevin Murphy, Cynthia Booth

**Affiliations:** University of Arizona College of Medicine Phoenix, Ringgold ID 42283, United States

**Keywords:** Postpartum hematoma, Vulvar hematoma, Non-resorbing, Delayed hematoma, Hematoma

## Abstract

Vulvar hematomas are more common in the obstetric population, and usually present within 24 h of delivery. Small, nonexpanding vulvar hematomas will often resolve with conservative management. In a rural setting in the USA, a 35-year-old woman, G3P3, presented to clinic 26 days after a home vaginal delivery attended by a midwife, which was complicated by postpartum hemorrhage secondary to retained placenta. Ten days after her delivery she developed intense pressure in her inferior right vulva. On examination a 4–5 cm well defined right vulvar mass was observed. Incision and drainage were performed and the mass was determined to be a hematoma that had not resorbed. Four days later, the patient returned to clinic as the mass had reformed. Computerized tomography did not show extravasation of contrast. As examination showed the mass was now 1 cm smaller, no intervention was undertaken and after one month the hematoma had completely resolved. This case provides a rare example of the delayed development of a vulvar hematoma. In the literature, the vast majority are reported to present within 24 h of delivery. Smaller hematomas, such as this one, which was 4-5 cm, are treated conservatively, as they typically absorb. This hematoma was present for approximately two weeks without resorbing.

## Highlights

• A 35-year-old parous woman developed a 4–5 cm vulvar hematoma 10 days after a home vaginal delivery attended by a midwife.
• She presented approximately two weeks later as the hematoma had not resorbed.
• In the literature, the vast majority of vulvar hematomas are reported to develop within 24 h of delivery.

## 1 Introduction

A hematoma is a collection of blood beneath an intact epidermis that presents as a swollen fluctuant lump which can be tender on palpation [1]. In the obstetric population, a vulvar hematoma most commonly results from direct or indirect injury to the soft tissue; the incidence is in the range of 1 hematoma per 300 to 1000 deliveries [2,3]. Small, nonexpanding vulvar hematomas will often resolve with conservative management. Here we describe a 4–5 cm vulvar hematoma that presented ten days after delivery and that did not resorb until well after two weeks.

## 2 Case Presentation

A 35-year-old woman, G3P3, presented to clinic as a referral from her midwife with concern for a Bartholin's gland cyst. She had had a vaginal delivery 26 days earlier, which had been complicated by postpartum hemorrhage secondary to retained placenta. Ten days after her delivery she developed intense pressure in her lower right vulvar area. It was not provoked and the patient denied any sexual intercourse, objects in the vagina, or any strenuous activity. She also denied any issues or complications after delivery. Since its development, she had felt it growing slowly until about a week prior to presentation, when believed it had stopped. It had not been draining any material. Prior to cessation of growth, she was beginning to feel pain in her rectum radiating towards her lower back.

**Fig. 1:**
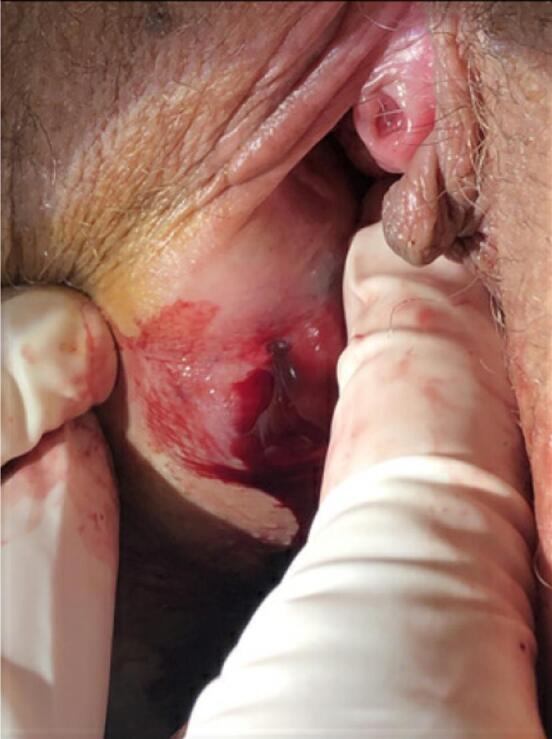
Hematoma presentation at initial visit

On physical exam, there was a 4–5 cm well circumscribed mass of moderate firmness at about 7–8 o'clock (using the vagina for reference) inside the tissue of the right vulva going inferiorly towards the right gluteal cleft. The mass was tender to palpation with no erythema to the surrounding area or discharge expressible from the mass. The mass felt mildly fluctuant. Palpation inward from the vagina indicated that the mass was limited to this area and did not track up the vagina or further into the perirectal space (see Fig. 1). As the area had been persistently expanding and had not resolved after almost 2 weeks, it was assumed that the mass was unlikely to be a hematoma. A decision for an incision and drainage procedure was made.

**Fig. 2:**
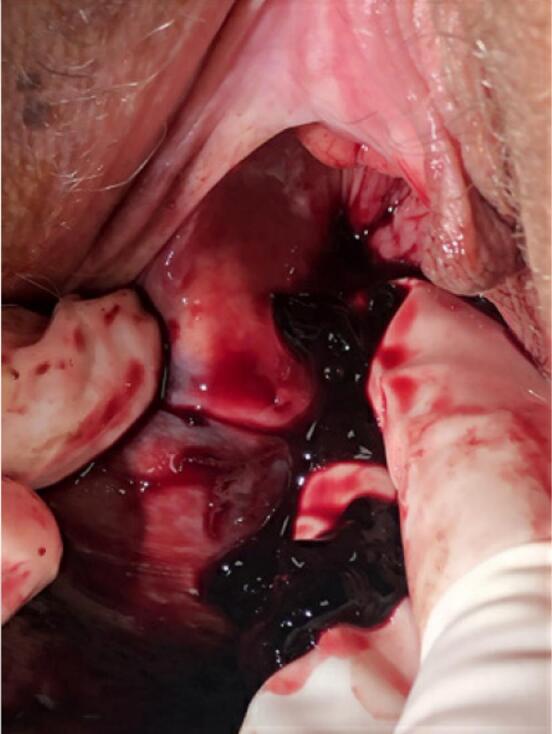
Hematoma after incision was made

The area was prepped with iodine solution and then anesthetized with 5 cc of 1% lidocaine with epinephrine. Using an 11-blade scalpel, a stab incision was made on the medial aspect of the mass. Instantly, dark, old-appearing blood came forth from the incision (see Fig. 2). Old blood was expressed but it was clear that there were other areas of the mass that required draining, so two more stab incisions were made in different aspects of the mass. The largest incision was no larger than 5 mm. It was clear after expressing from these incisions that this was an old hematoma that had not resorbed. After complete expression of the mass the incisions were irrigated and then cleaned again with iodine. Two of the incisions were closed with 4–0 Monocryl in a single subcuticular stitch. The third, the smallest of the three, was left open to allow continuous drainage. All sites were hemostatic at the end of the procedure. The area was cleaned with gauze soaked in sterile water. At conclusion of the procedure the mass was not reforming and there was no concern for the hematoma tracking in a new path at that time.

Four days after this drainage, the mass had reformed so the patient underwent computerized tomography with contrast, which did not show any extravasation.

Three days later, the patient returned to clinic to report that she still had pressure in the area but it was less than before. On physical exam, the hematoma was smaller than before by about 1 cm in all dimensions, with no signs of infection. Due to the hematoma being smaller, it was decided that did not need to be explored surgically. One month later, the patient returned for a second follow-up. On physical exam, the hematoma had completely resolved and patient had no additional concerns.

## 3 Discussion

A hematoma is defined as a collection of blood beneath an intact epidermis that presents as a swollen fluctuant lump which can be tender on palpation [1]. A vulvar hematoma is thus a collection of blood in the vulva. The pudendal artery is the main supply of blood to the vulva [4]. Most commonly, a vulvar hematoma in the obstetric population is the result of direct or indirect injury to the soft tissue, and the incidence is in the range of 1 per 300 to 1000 deliveries [2,3]. Examples of direct soft-tissue injuries include lacerations, episiotomy, or operative delivery while extensive stretching of the birth canal during vaginal delivery is an example of an indirect injury [5].

The median time to detect formation of a hematoma is 6 h postpartum [6], while the most common presenting sign of a hematoma is pain within 24 h of delivery [2,3]. One retrospective study found that 46.2% of puerperal hematomas were associated with severe pain, and 61.5% of the hematomas were detected within 2 h of delivery [7]; 61.5% of cases had the hematoma on the right side [7]. Delayed detection of a vascular injury could be due to pressure necrosis, while immediate detection is seen with direct vessel injury [8]. The risk factors for formation of a vulvo-vaginal hematoma include hypertension, multiple gestation, infants that are large for gestational age, lacerations, clotting abnormalities, episiotomy, larger head circumference, vulvovaginal varicosities, and prima-gravidity [9,10]. The authors' literature search identified only one earlier report (from 2019) of a postpartum hematoma that formed over a week (10 days) after delivery [11]. In 2013 a case was reported of a vulvovaginal thrombus that occurred 5 days postpartum [12]. Though no studies were found comparing vulvar hematoma rates of home vs hospital delivery, home deliveries are associated with increased adverse events, including perinatal deaths and seizures/neurologic problems [13,14].

There is limited data on the optimal management of vulvar hematoma [3]. Small, nonexpanding vulvar hematomas will often resolve without intervention, and conservative management will avoid introducing bacteria and as well as potentially difficult and unnecessary surgical procedures [15]. Examples of conservative management include analgesia, sitz baths, empiric antibiotics, pain medication and application of cold packs [16,17]. The rationale for conservative management is that soft-tissue swelling and space limitations will result in tamponade of bleeding vessels [18]. Conservative management involves minimal additional treatment measures [15]. One retrospective study found that 53.8% of hematomas were ≥ 5 cm in size [7]. In contrast, larger (>12 cm) hematomas, continuously expanding hematomas, or those large enough to cause either urologic or neurologic symptoms may require management with surgical exploration or vessel ablation through interventional radiology. One retrospective study found that 61.5% of detected hematomas required surgical treatment [7]. In the majority of cases, evacuation, drainage and ligation of bleeding vessels are sufficient to achieve hemostasis [15].

## 4 Conclusion

This case provided a rare example of a vulvar hematoma that presented over a week postpartum. As seen through the literature search, the vast majority of postpartum hematomas present within 24 h of delivery, with a median time of 6 h. Smaller hematomas, such as this one, which was 4–5 cm, are treated conservatively, as they typically resorb. This hematoma was present for approximately two weeks without resorbing.
